# Rehabilitation after supracricoid partial laryngectomy: cohort study

**DOI:** 10.1016/j.bjorl.2024.101532

**Published:** 2024-11-19

**Authors:** Caroline da Silva Seidler, Marianne Yumi Nakai, Lucas Ribeiro Tenório, Daniela Serrano Marquezin, Renata Santos Bittencourt Silva, Marcelo Benedito Menezes, Antonio José Gonçalves

**Affiliations:** Irmandade Santa Casa de Misericórdia de São Paulo, São Paulo, SP, Brazil

**Keywords:** Laryngeal neoplasms, Laryngectomy, Rehabilitation of speech and language disorders, Rehabilitation, Quality of life

## Abstract

**Objectives:**

To evaluate vocal, swallowing and respiratory rehabilitation of patients undergoing supracricoid laryngectomy; evaluate the impact of voice changes and global quality of life.

**Methods:**

It is a prospective cohort study where voice, swallowing and respiratory rehabilitation where evaluated. Quality of life was assessed using EORTC QLQ-C30 and H&N35 questionnaires.

**Results:**

31 patients were included in the study. 70.4% of patients were T3. In swallowing assessment, two patients (6.4%) were considered non-rehabilitated. Ten patients (32.2%) were considered partially rehabilitated and 19 (61.3%) fully rehabilitated. Sensitivity was abnormal at 74.2%. Penetration occurred in 58.1% of patients with 29% experiencing aspiration. In voice assessment, 19 (61.3%) patients were considered rehabilitated. 77.41% had a calculated voice handicap index considered abnormal. When assessing breathing, eight patients (25.8%) were considered non-rehabilitated. Disease-free survival was 87.1% in follow-up of 5.77 years. When subjected to specific exams and evaluations, only 38.7% were completely rehabilitated. When comparing results of EORTC-C30 and H&N35 questionnaires with the reference values, global score and domain scores are better on the patients of the study, as well as some of the symptoms.

**Conclusion:**

Partial supracricoid laryngectomy remains an option in patients with laryngeal tumors, especially in the most advanced ones, with a survival rate around 90%. Quality of life is impacted after surgery, but with few symptoms.

**Level of evidence:**

Level 3.^1^

## Highlights

• Supracricoid laryngectomy remains a good option for laryngeal tumors.
• Moderate to severe vocal disability is expected, but a great quality of life remains.
• Patients previously rehabilitated experienced aspiration with specific exams.
• Penetration and aspiration do not worsen quality of life and survival.
• Oncological results are encouraging, with an average overall survival around 79.7%.

## Introduction

Head and neck squamous cell carcinoma represent around 4.6% of death from cancer worldwide.^2^ Supracricoid Partial Laryngectomy (SCPL) was first described in 1959 by Majer and Rieder for treatment of laryngeal carcinoma. It was modified by Piquet et al. in 1974. In the 1990s Laccourreye and collaborators spread its use for treatment of glottic cancer throughout Europe.^3^

Advantages compared to total laryngectomy are absence of a permanent tracheostoma and retention of physiological voice and swallowing.^4^ Oncological results are encouraging, with an average overall survival around 80%.^5^, ^6^

Rehabilitation after surgery aims to optimize function of remaining structures in order to achieve the best of these impaired functions.^7^ Through clinical examinations, swallowing has good rehabilitation rates, however, when a targeted examination is carried out, aspiration is present in around 34% of patients.^8^, ^9^ Voice is audible and understandable, but a prolonged period of rehabilitation is necessary.^10^, ^11^

Efforts are being made to identify the impact of this surgery on patients' quality of life.^12^, ^13^ Even though it is believed that patients undergoing partial surgery have greater difficulty eating, this difficulty does not seem to have a significant impact on their quality of life.^14^

In addition, self-assessment of how much a voice problem can affect quality of life provides important data for diagnosing voice quality. There are new instruments validated for this purpose, such as the Vocal Handicap Index (VHI-10).^15^, ^16^

Study's main objective is to evaluate vocal, swallowing and respiratory rehabilitation of patients undergoing Supracricoid Laryngectomy (SCPL) in Head and Neck Surgery Departament of Irmandade Santa Casa de Misericórdia de São Paulo (ISCMSP). Among secondary objectives are: (1) Evaluate impact of voice changes in these patients, using VHI-10; (2) Evaluate global quality of life of patients undergoing SCPL.

## Methods

It’s a prospective cohort study with patients undergoing SCPL from February 2004 to June 2021. Inclusion criteria were: (1) Patients undergoing SCPL in the service in question; (2) Minimum postoperative period of 6 months; (3) Minimum time of 4 months in speech therapy. Exclusion criteria were: (1) Patient with tumor recurrence; (2) Active oncological disease; (3) Death; (4) Patients undergoing salvage surgery after radiotherapy.

Sampling was for convenience, where medical records and face-to-face interview data were used. Postoperative assessments were carried out between January 2020 and June 2022. Recruited patients signed a free informed consent form.

Patients were operated by resident doctors, under a senior surgeon supervision. On admission day for surgery, patients underwent an interview with a specialized speech therapist. Tracheostomy cuff was generally deflated o 3rd post-operative day and cannula was changed for a metal one between 4th and 5th post-operative day. Patients were discharged using a nasoenteral tube, without oral feeding. Return to outpatient clinic generally occurred 7–14 days after surgery and evaluation by a specialized speech therapist began approximately 15 days after surgery.

Patients with indication for adjuvant radiotherapy remained using tracheostomy until treatment was completed due to risk of acute respiratory failure and difficulty in accessing urgent and emergency services with qualified staff.

Demographic and surgical data, medical history and lifestyle habits were collected from patient's medical records and during interview. All patients were considered to have previously rehabilitated their larynx primary functions: swallowing, speaking and breathing. Indication for radio/chemotherapy followed NCCN® criteria, considering adverse factors such as close or positive margins, extranodal extension, pT3 or pT4, pN2 or pN3, perineural or angiolymphatic invasion and subglottic extension.

All questionnaires and assessments were stored in the REDCap database.

For voice assessment, a recording was made on a cellphone positioned 10 cm from patient's mouth and a speech sample was obtained through reproduction of the following tasks: (1) Emission of sustained vowel /a/; (2) Free speech: patient responded to the question: “How did he/she get here to the hospital?”

Patients also responded a questionnaire on self-perception of vocal quality (Vocal Handicap Index ‒ VHI-10), an instrument that contains 10 self-evaluative questions, answered on a 5-point scale, with 0 never and 4 always. It was standardized that values above 11 should be considered as abnormal VHI-10.^17^

Following data were evaluated to characterize vocal rehabilitation:

- Sound emission intensity of vowel /a/: measured in decibels using a SMART SENSOR brand decibel meter positioned 10 cm from patient;
- Maximum Phonation Time (MPT) in seconds: subjects were asked to emit sustained vowel /a/ in a single exhalation. Three attempts were made, and the highest value obtained was considered^18^;
- Auditory-perceptual analysis: recorded voice materials were sent for analysis of three speech therapists specialized in head and neck rehabilitation. Voices were classified using GRBASI scale, with the aim of classifying the degree of dysphonia (G – Global, R – Roughness, B – Breathiness, A – Asthenia, S – Strain, I – Instability).^18^, ^19^, ^20^

Rehabilitation was complete if two of the three speech therapists considered the patient rehabilitated.

To evaluate swallowing rehabilitation, patients underwent nasofibroscopy with Pentax3.5 mm flexible fiber optics. Pasty, liquid and solid consistencies with pigment were offered. The resulting video was evaluated by an otolaryngologist.

During staccato and blowing vowels, nasopharynx was evaluated for presence of asymmetries and mobility of soft palate and lateral wall, also evaluating adequate closure of the soft palate; salivary stasis was assessed based on location (valecula, pharynx, supraglottis or glottis, recesses) and degree of stasis.

Patient was asked to hold the food in mouth for 3 seconds before swallowing, where the presence or absence of escape was assessed. As in studies conducted by Freitas et al.,^8^, ^9^ penetration was considered when food contact with remnants of supraglottic structure (level of the neoglottis) without exceeding level of the base of remaining arytenoid and aspiration was considered when food exceeded level of the remaining arytenoid.

Patients were considered non-rehabilitated when there was aspiration in all consistencies; partially rehabilitated when aspiration occurred in one or two consistencies offered; and completely rehabilitated when there was no aspiration.

Patients who removed tracheostomy was considered rehabilitated from a respiratory point of view, regardless of the time.

Quality of life was assessed using the EORTC QLQ-C30 version 3.0 and H&N35 questionnaires. Score was calculated according to instructions in the EORTC QLQ-C30 Scoring Manual^21^ and compared with reference values observed by the organization itself.^22^ Higher scores on a functional scale represent a high/healthy level of functioning, while a high score for a scale or item for symptoms represents a high level of symptomatology or problems.^21^, ^23^ H&N35 Appendix was developed specifically for head and neck cancer patients, with 35 symptom-related questions and scoring approach is identical in principle to that for QLQ-C30 single-item/symptom scales.^24^

Descriptive statistics was used to desbribe baseline characteristics and other variables results. Continuous variables were reported with central tendency measures, such as mean and standard deviation, while categorical variables were presented as frequency and percentage.^25^

To assess relationship between predictor variables and outcomes, a logistic regression model was performed for each outcome. The choice of covariables was based on previous literature, clinical relevance and statistical significance in univariate analysis. For univariate analysis, the normality test was performed in all continuous variables using Kolmogorov–Smirnov test.^26^ Chi-Square was used to categorial variables and Student's *t*-test or Mann–Whitney *U* test for continuous variables, as appropriate. Level of significance was set at *p*-value < 0.05. Addicionally, the Hosmer and Lemeshow technique was used to improve model.^27^

All statistical tests were performed using STATA BE v.18 for Mac software.

## Results

Thirty-one patients were included in the study, 24 male (77.4%) and 7 female (22.6%). Average age at surgery was 60 years. Hypertension was the most prevalent comorbidity (63.2%), followed by diabetes mellitus (10.5%). Twelve patients (38.7%) reported alcohol consumption and 27 patients (87.1%) reported smoking.

Surgery performed in all cases was supracricoid laryngectomy. One patient underwent SCPL as salvage surgery due to a second primary after 7 years, with frontolateral partial laryngectomy performed as previous treatment.

Most prevalent subsite was glottis 27 (87.1%), with two cases of hypopharynx (6.5%) and two of supraglottis (6.5%). The average length of stay after procedure was 10.5 days.

In 25 cases (80.6%) one arytenoid was preserved. Preservation of epiglottis ocurred in 24 cases (77.4%). Neck dissection was unilateral in 22 patients (71%) and bilateral in eight cases (25.8%).

Final staging of two patients with supraglottis tumors was T2N0 and T3N2a. One (50%) required adjuvant radiotherapy. Final staging of two patients with hypopharyngeal tumors was T2N3b and T3N3a. One (50%) underwent exclusive adjuvant radiotherapy and one (50%) underwent chemotherapy and radiotherapy.

Demographic data of patients submitted to study are described in Table 1. Table 2 shows characterization by surgical and anthropometric data. Patients with supraglottic and hypopharyngeal tumors were omitted due to limited sample size, which made statistical analysis impossible. In univariate analysis, only ECOG scale (*p* = 0.001), preservation of epiglottis (*p* = 0.048), post-chemotherapy (*p* = 0.042) and concomitant radio-chemotherapy (*p* = 0.042) were statistically significant for rehabilitation. However, in multivariate analysis, this was not confirmed.

**Table 1:** Sample characterization ‒ demographic data (n = 27).

				Partially		Not				
		Rehabilitated (n = 11)		Rehabilitated (n = 15)		Rehabilitated (n = 1)		Total		*p*
Age (average, in years)		60.1		65.5		61		63.78		0.330
Gender (no, %)	Male	8	72.3%	11	73.3%	1	100%	20	74%	0.883
	Female	3	27.2%	4	26.6%	0	0	7	26%	
Scholarity (no, %)										0.147
	Incomplete fundamental school	9	81.8%	11	73.3%	0	0	20	74%	
	Complete fundamental school	1	9.1%	0	0	0	0	1	3.7%	
	Incomplete high school	1	9.1%	1	6.7%	1	100%	3	11.1%	
	Completed high school	0	0	2	13.3%	0	0	2	7.4%	
	Graduated at university	0	0	1	6.7%	0	0	1	3.7%	
Self-designation (no, %)	Black	1	9%	1	6.7%	0	0	2	7.4%	0.924
	Brown	6	55.6%	8	53.3%	1	100%	15	55.6%	
	White	4	36.4%	6	40%	0	0	10	37%	
ECOG scale (no, %)	0	10	90.9%	15	100%	0	0	25	92.6%	**0.001**
	1	1	9.1%	0	0	1	100%	2	7.4%	
Religion (no, %)	Catholicism	7	63.6%	10	66.7%	1	100%	18	66.7%	0.845
	Evangelism	3	27.3%	4	26.7%	0	0	7	25.9%	
	Atheism	1	9.1%	0	0	0	0	1	3.7%	
	Others	0	0	1	6.7%	0	0	1	3.7%	
Work situation (no, %)	Retired	3	27.3%	9	60%	1	100%	13	48.1%	0.834
	Unemployed	2	18.2%	2	13.3%	0	0	4	14.8%	
	Regular job	2	18.2%	1	6.7%	0	0	3	11.1%	
	Freelance professional	2	18.2%	2	13.3%	0	0	4	14.8%	
	Do not work	2	18.2%	1	6.7%	0	0	3	11.1%	
Income (no, %)	Up to 2 minimum wage	9	81.8%	7	46.7%	1	100%	17	63%	0.543
	2 to 4 minimum wage	1	9.1%	6	40%	0	0	7	25.9%	
	4 to 10 minimum wage	1	9.1%	1	6.7%	0	0	2	7.4%	
	uninformed	0	0	1	6.7%	0	0	1	3.7%	
Comorbidities (no, %)	No	4	36.4%	5	33.3%	1	100%	10	37%	0.409
	Yes	7	63.6%	10	66.7%	0	0	17	63%	

Note: for this table, patients with tumors located in the glottis were considered. Patients with hypopharyngeal and supraglottis tumors were omitted due to their limited sample size, which made statistical analysis impossible.

**Table 2:** Sample characterization ‒ surgical and anthropometric data (n = 27).

				Partially						
		Rehabilitated (n = 11)		Rehabilitated (n = 15)		Not rehabilitated (n = 1)		Total		*p*
Nasoenteral tube time (average, in months)		4.5		4.27		‒		5.15		0.272
Tracheostomy time (average, in months)		9.9		24.72		‒		10.75		0.142
Muscle mass (average, in %)		32.5		30.75		‒		30.96		0.297
Length of stay (average, in days)		8.9		8.09		27		9.76		0.629
BMI		26.1		25.1		16.2		24.51		0.289
Arytenoid preservation (no, %)	One	9	81.8%	13	86.7%	1	100%	23	85.2%	0.861
	Both	2	18.2%	2	13.3%	0	0	4	14.8%	
Epiglottis preservation (no, %)	Type IIa (CHEP)	10	90.9%	13	86.7%	0	0	23	85.2%	**0.048**
	Type IIb (CHP)	1	9.1%	2	13.3%	1	100%	4	14.8%	
Complications (no, %)	No	6	54.5%	4	26.7%	0	0	10	37%	0.256
	Yes	5	45.4%	11	73.3%	1	100%	17	63%	
Post chemotherapy (no, %)	No	8	72.7%	14	93.3%	0	0	22	81.5%	**0.042**
	Yes	3	27.3%	1	6.7%	1	100%	5	18.5%	
Post radiotherapy (no, %)	No	6	54.5%	9	60%	0	0	15	55.6%	0.503
	Yes	5	45.5%	6	40%	1	100%	12	44.4%	
Concomitant QT/RT (no, %)	No	8	72.7%	14	93.3%	0	0	22	81.5%	**0.042**
	Yes	3	27.3%	1	6.7%	1	100%	5	18.5%	
Final T staging (no, %)	T2	3	27.3%	5	33.3%	0	0	8	29.6%	0.706
	T3	8	72.7%	10	66.7%	1	100	19	70.4%	
Final N staging (no, %)	N0	9	81.8%	14	93.3%	1	100%	24	88.9%	0.792
	N1	1	9.1%	1	6.7%	0	0	2	7.4%	
	N2b	1	9.1%	0	0	0	0	1	3.7%	

Note: for this table, patients with tumors located in the glottis were considered. Patients with hypopharyngeal and supraglottis tumors were omitted due to their limited sample size, which made statistical analysis impossible.

Twenty (64.5%) patients developed postoperative complication. Six (30%) were early, occurring within 15 days of the surgical procedure: one pneumonia, two nerve paresis, one wound infection, one bleeding at first post-operative day and one pharyngocutaneous fistula. Fourteen (70%) were late ones, 15 days after surgery: one aspiration pneumonia, four tracheocutaneous fistulas, three mucosal redundancies, one deep vein thrombosis, one laryngeal collapse, one neoglottis stenosis, two bleedings and two pharyngocutaneous fistulas.

Average length of stay with nasoenteral tube was 5.4 months. Two patients required gastrostomy, but by the time of the study, were eating orally.

### Swallowing assessment

Due to massive aspiration during ingestion of soft food in two patients, it was decided not to offer liquid or solid food in these cases. These two patients (6.4%) were considered non-rehabilitated. Ten patients (32.2%) were considered partially rehabilitated and 19 (61.3%) fully rehabilitated.

Results are exposed in Table 3, Table 4.

**Table 3:** Swallowing assessment using flexible nasofibroscopy (n = 31).

		n	%
Hypernasality	Light	3	9.7
	Moderate	1	3.2
	Serious	0	0
	No	27	87.1
Nasopharyngeal asymmetry	Yes	1	3.2
	No	30	96.8
Palate mobility	Yes	31	100
Side wall mobility	Yes	30	96.8
	No	1	3.2
Velopharyngeal function ‒ murmur	Normal	28	90.3
	Abnormal	3	9.7
Velopharyngeal function ‒ staccato vowel	Normal	26	83.9
	Abnormal	5	16.1
Velopharyngeal function ‒ swallowing	Efficient	29	93.5
	Inefficient	2	6.5
Salivary stasis	Vallecula	5	16.1
	Pharynx	2	6.5
	Supraglottis/Glottis	1	3.2
	Recesses	8	25.8
	Penetration	1	3.2
	No	14	45.2
Sensitivity	Normal	8	25.8
	Abnormal	23	74.2
Nasal reflux	Light	4	12.9
	Moderate	0	0
	Serious	1	3.2
	No	26	83.9

**Table 4:** Assessment after swallowing during flexible nasofibroscopy.

		Pasty (n = 31)		Liquid (n = 29)		Solid (n = 29)	
Number of swallows (mean, median)		3.19	3	2.34	2	2.41	2
Stasis (no, %)	Light	6	19.4	11	37.9	8	27.6
	Moderate	13	41.9	2	6.9	4	13.8
	Serious	2	6.5	0	0	0	0
	No	10	32.3	16	55.2	17	58.6
Escape (no, %)	Yes	3	9.7	5	17.2	7	24.1
	No	28	90.3	24	82.8	22	75.9
Penetration (no, %)	Yes	18	58.1	16	55.2	12	41.4
	No	13	41.9	13	44.8	17	58.6
Aspiration (no, % )	Yes	9	29	3	10.3	4	13.8
	No	22	71	26	89.7	25	86.2
Cough (no, %)	Yes	10	32.3	8	27.6	5	17.2
	No	21	67.7	21	72.4	24	82.8

Note: Two patients had significant aspiration in the test with pasty food and therefore did not carried out tests with liquids and solids.

### Voice assessment

The average value reached during sustained /a/ vowel was 74.1 decibels and maximum phonation time was 4.20 seconds. Table 5 summarize GRBASI scale and perception of speech therapists regarding rehabilitation. Nineteen (61.3%) patients were considered rehabilitated.

**Table 5:** GRBASI assessment and vocal rehabilitation (n = 31).

		Speech therapist 1		Speech therapist 2		Speech therapist 3	
		n	%	n	%	n	%
Grade	Moderate	22	71	11	35.5	4	12.9
	Severe	9	29	20	64.5	27	87.1
Roughness	Normal/absent	0	0	4	12.9	0	0
	Discreet	8	25.8	0	0	0	0
	Moderate	15	48.4	11	35.5	4	12.9
	Severe	8	25.8	16	51.6	27	87.1
Breathiness	Normal/Absent	0	0	21	67.7	0	0
	Discreet	17	54.8	3	9.7	0	0
	Moderate	11	35.5	3	9.7	7	22.6
	Severe	3	9.7	4	12.9	24	77.4
Asthenia	Normal/Absent	28	90.3	26	83.9	31	100
	Discreet	3	9.7	1	3.2	0	0
	Moderate	0	0	3	9.7	0	0
	Severe	0	0	1	3.2	0	0
Strain	Normal/Absent	0	0	14	45.2	0	0
	Discreet	4	12.9	8	25.8	0	0
	Moderate	23	74.2	9	29	4	12.9
	Severe	4	12.9	0	0	27	87.1
Instability	Normal/Absent	1	3.2	21	67.7	1	3.2
	Discreet	16	51.6	7	22.6	0	0
	Moderate	14	45.2	3	9.7	11	35.5
	Severe	0	0	0	0	19	61.3
Rehabilitated	Yes	18	58.1	16	51.6	26	83.9
	No	13	41.9	15	48.4	5	16.1

Seven patients (22.58%) had a calculated VHI ≤ 11 and 24 (77.41%) had a calculated VHI greater than 11 and were therefore considered abnormal.

### Breathing assessment

Twenty-three patients (74.2%) were rehabilitated and average length of stay with tracheostomy in these patients were 10.9 months.

### General rehabilitation and follow-up

Table 6 summarizes rehabilitation rates.

**Table 6:** Rehabilitation rate of study patients (n = 31).

	Fully Rehabilitated		Partially Rehabilitated		Not rehabilitated	
	n	%	n	%	n	%
Final rehabilitation	12	38.7	17	54.8	2	6.5
Swallowing rehabilitation	19	61.3	10	32.2	2	6.5
Voice rehabilitation	19	61.3	‒	‒	12	38.7
Breathing rehabilitation	23	74.2	‒	‒	8	25.8

Follow-up was 5.77 years (1–18 years). 27 patients (87.1%) were alive and disease-free at the end of study. One patient (3.2%) with a glottic tumor underwent laryngeal totalization due to dysfunction and one (3.2%) due to recurrence. Two patients (6.5%) died from the disease, one with lymph node recurrence and the other with recurrence in primary site.

### Quality of life

Data obtained and calculated according to the EORTC C30 and H&N35 manual are described in Table 7, Table 8.

**Table 7:** General quality of life result - EORTC QLQ C30 (n = 31).

		Average^b^	Reference value^a^
Global quality of life		84.97	62.3
Funcional	Physical	80.23	73
	General	86	72.8
	Emotional	77.74	69.4
	Cognitive	91.35	83.7
	Social	88.13	78.5
Symptoms	Fatigue	18.97	32
	Nausea and vomiting	7.55	5.3
	Pain	14.52	22.3
	Shortness of breath	22.58	28.4
	Insomnia	21.45	27.7
	Lack of appetite	17.23	15.4
	Constipation	6.45	7.5
	Diarrhea	3.23	7.6
	Financial difficulties	27.97	24.8

a Reference values were obtained according to the EORTC reference value.^19^

b Quality of life was measured by a scale from 0 to 100 in which higher scores on a functional scale represent a high/healthy level of functioning, while a high score for a symptom scale or item represents a high level of symptomatology or problems.

**Table 8:** Quality of life results specific to head and neck ‒ EORTC H&N35 (n = 31).

	Average^b^	Reference value^a^
Pain	11.42	35.3
Swallowing problems	22.58	36.7
Sensory problems	11.77	38.7
Problems with speech	36.48	50
Problems eating in public	14	32.4
Problems with social contact	10.84	28.3
Reduced sexuality	18.77	46.8
Dentition	9.68	36.5
Mouth opening	5.35	30.5
Dry mouth	36.55	44.1
Thick saliva	40.90	45
Cough	24.68	54.8
Feeling sick	12.90	40.9
Use of pain killers	45.16	71.5
Use of dietary supplement	25.81	61
Feeding device	6.45	58
Weight loss	19.35	67.1
Weight gain	44.48	58

a Reference values were obtained according to the EORTC reference value.^19^

b Quality of life was measured by a scale from 0 to 100 in which higher scores on a functional scale represent a high/healthy level of functioning, while a high score for a symptom scale or item represents a high level of symptomatology or problems.

## Discussion

Most cases were advanced tumors ‒ 70.4% of patients were T3. 85.2% preserved epiglottis with Crico-Hyoido-Epiglottopexy (CHEP) reconstruction. Complications, although minor, accounted for 63%. Disease-free survival was 87.1% in follow-up average of 5.77 years.

Salivary stasis was presented in 54.8% of all cases. Sensitivity was abnormal at 74.2%. Penetration occurred in 58.1% of cases with 29% experiencing aspiration. All patients had a moderate to severe degree of vocal disability and 77.41% had a calculated VHI greater than 11, considered abnormal. 25.8% of patients remained with a tracheostomy tube.

When crossing evaluation of the three functions of the larynx: voice, swallowing and breathing), only 2 patients (6.5%) were considered not rehabilitated, although they were previously cleared by speech therapy through clinical analysis. When subjected to specific exams and evaluations, only 38.7% were completely rehabilitated.

When comparing results of EORTC-C30 and H&N35 questionnaires with reference values, global score and domain scores are better on patients of the study, as well as some of symptoms. However, financial impact is greater than the reference value. These are patients with low financial conditions and a lower level of education, which may corroborate with this result.

Nasoenteral tube removal and decannulation time are commonly used to evaluate functional outcomes of SCPL.^28^ A large systematic review carried out by Schindler et al.^29^ showed that average hospital stay varied from 5 days^30^ to 104 days.^31^ Feeding tube removal varied between 10–88 days, and a safe and unrestricted oral diet was achieved by 53%–100% of patients within first postoperative year. Great heterogeneity was found in average decannulation time, varying between 8–105 days. On the other hand, little variability was found in decannulation rates, which range between 85.7% and 100%.^29^

In the present study, average length of stay was 10.52 days, consistent with studies presented. Average length of stay for nasoenteral tube was 5.4 months (162 days), higher in relation to data provided by authors. Average length of stay with tracheostomy was 10.9 months, also increased in relation to other studies, with decannulation rate being 74.2%. This may be due to lack of adequate speech therapy. Furthermore, these are patients with a low level of education, which often makes it difficult to understand the exercises necessary for rehabilitation and their importance.

There is also no consensus on factors that would influence time taken to remove tracheostomy and nasoenteral tube, or vocal quality of patients, with authors finding that resection of an arytenoid or resection of epiglottis could compromise rehabilitation^28^, ^32^ and others without evidence that removal of structures or pre-operative radiotherapy have influenced the results.^11^, ^33^, ^34^

Webster et al.^4^ found that one year after surgery, approximately 80% of patients safely ingested all consistencies, but 67% (6 of 9) aspirated silently at some point during examination, a fact also described by Zacharek et al.,^34^ where interestingly, none of the patients were hospitalized for aspiration pneumonia in postoperative period. Freitas et al.^9^ also noted that, although inclusion criterion for study was complete rehabilitation, 68.6% of patients had laryngeal penetration and 34.3% had laryngotracheal aspiration. These findings would suggest that all patients had adequate cough reflexes and a functioning tracheopulmonary mucociliary clearance system, which helped protect the lungs from aspirated material, a fact corroborated by other authors.^26^, ^29^

In the present study, although all patients were also considered rehabilitated from swallowing point of view, laryngeal sensitivity was altered in 74.2% of patients, penetration was present in 58.1% and aspiration in 29%, with only 1 patient (3.22%) presenting with late aspiration pneumonia. This corroborates the literature.

Only a few studies have focused on voice characteristics in patients after SCPL, finding a highly reduced MPT, with values ranging between 8 and 11 seconds.^28^, ^30^ It confirms that there is a significant loss of air during phonation after surgery because of incomplete neoglottis closure. Furthermore, in the studies, all patients were perceived by experienced evaluators as having a moderate to severe degree of a hoarse and breathy vocal quality.^5^, ^35^

In this study, MPT was 4.2 s, even worse than established in literature and as seen, VHI was abnormal in 77.41% of patients, showing that there is an important loss in vocal quality. Speech therapists' perception was also of a hoarse voice in majority of patients in a moderate to severe degree, in agreement with literature, although perception of each judge speech therapist varied widely. This discrepancy between evaluators is explained by the subjectivity of the GRBASI scale, which considers perception of each evaluator, although they all trained in the same reference center.

Perhaps start of speech therapy in this study was late and vocal results would be better if patients were evaluated and advised prior to surgery and started therapy on first day after surgery. Such logistics is still difficult at the institution. Removing tracheostomy later can also be a complicating factor.

Complication rates vary in the literature.^20^, ^31^, ^32^ In the study in question, rates are considered high, with 20 (64.5%) patients presenting some type of complication. As it is a health center that generally serves a poorer population, perhaps the degree of malnutrition in preoperative period could contribute to these higher rates.

When analyzing quality of life, some studies have observed that although there is a clear loss of voice quality, patients present relatively satisfactory emotional, physical and functional levels, suggesting that oral communication was not significantly limited.^14^, ^36^, ^37^, ^38^

Mesolella et al.^35^ also used the EORTC-30/H&N35 to evaluate quality of life of its patients undergoing SCPL and recorded that in general, patients fell into medium or low range (less than 50%), which corresponds to a better health condition perceived by the patient. Among other factors that worsen quality of life, there are swallowing problems and difficulties in social relationships.

It is interesting to note that recurrence rate found in the study is similar to literature, with 9.6% recurrence/deaths due to disease and a disease-free survival rate of 90.3%.^5^, ^6^, ^35^, ^39^

The main limitation of the present study is represented by the small sample size and the fact that it is a convenience sample, not random, of a single hospital. Furthermore, patients were not operated by the same surgeon. These aspects as limitations and can generate biases.

SCPL has good rehabilitation and disease-free survival rates. It´s important that complete rehabilitation could be evaluated using already established parameters, but also through complementary exams, such as nasofibroscopy, when available. Although there are no direct complications, such as aspiration pneumonia, some degree of dysphagia is observed on targeted examination. Vocal quality is altered, with an impact on voice perception in these patients, who notice this change, but maintain good levels of overall quality of life. It is still necessary to understand which possible predictors lead to better rehabilitation so indication could be more accurate. To this end, new studies with greater casuistic and targeted must be promoted.

## Conclusion

Under the conditions of the present study, partial supracricoid laryngectomy remains an option in patients with laryngeal tumors, especially in the most advanced ones (70% of the patients in the study were T3), with survival rates around 90%. Quality of life is impacted after surgery, but with few symptoms.

Future studies focused on this topic should define clinical and functional predictive factors of recovery in order to guide doctors in the surgical planning process.

## Funding

This research did not receive any specific grant from funding agencies in the public, commercial, or not-for-profit sectors.

## Declaration of competing interest

The authors declare no conflicts of interest.
